# Structural Changes of the Paraflagellar Rod during Flagellar Beating in *Trypanosoma cruzi*

**DOI:** 10.1371/journal.pone.0011407

**Published:** 2010-06-30

**Authors:** Gustavo Miranda Rocha, Dirceu Esdras Teixeira, Kildare Miranda, Gilberto Weissmüller, Paulo Mascarello Bisch, Wanderley de Souza

**Affiliations:** 1 Laboratório de Ultraestrutura Celular Hertha Meyer, Universidade Federal do Rio de Janeiro, Cidade Universitária, Ilha do Fundão, Rio de Janeiro, Brazil; 2 Instituto Nacional de Ciência e Tecnologia em Biologia Estrutural e Bioimagens, Universidade Federal do Rio de Janeiro, Cidade Universitária, Ilha do Fundão, Rio de Janeiro, Brazil; 3 Diretoria de Programa, Instituto Nacional de Metrologia, Normalização e Qualidade Industrial - INMETRO, Duque de Caxias, Rio de Janeiro, Brazil; 4 Laboratório de Física Biológica, Instituto de Biofísica Carlos Chagas Filho, Universidade Federal do Rio de Janeiro, Cidade Universitária, Ilha do Fundão, Rio de Janeiro, Brazil; State University of Campinas (UNICAMP), Brazil

## Abstract

**Background:**

*Trypanosoma cruzi*, the agent of Chagas disease, is a protozoan member of the Kinetoplastidae family characterized for the presence of specific and unique structures that are involved in different cell activities. One of them is the paraflagellar rod (PFR), a complex array of filaments connected to the flagellar axoneme. Although the function played by the PFR is not well established, it has been shown that silencing of the synthesis of its major proteins by either knockout of RNAi impairs and/or modifies the flagellar motility.

**Methodology/Principal Findings:**

Here, we present results obtained by atomic force microscopy (AFM) and transmission electron microscopy (TEM) of replicas of quick-frozen, freeze-fractured, deep-etched and rotary-replicated cells to obtain detailed information of the PFR structures in regions of the flagellum in straight and in bent state. The images obtained show that the PFR is not a fixed and static structure. The pattern of organization of the PFR filament network differs between regions of the flagellum in a straight state and those in a bent state. Measurements of the distances between the PFR filaments and the filaments that connect the PFR to the axoneme as well as of the angles between the intercrossed filaments supported this idea.

**Conclusions/Significance:**

Graphic computation based on the information obtained allowed the proposal of an animated model for the PFR structure during flagellar beating and provided a new way of observing PFR filaments during flagellar beating.

## Introduction

Much has been learned in the last decade on the structural organization of cells, mainly due to the development of and improvement in molecular, biochemical and microscopy methods. In the case of protozoa of the Trypanosomatidae family, which comprise important agents of human and animal diseases, the organization of cells has been previously analyzed in some detail using a combination of transmission electron microscopy (TEM) methods, such as observation of thin sections as well as replicas obtained after freeze-fracture [1]. The use of quick-freezing, freeze-fracture, deep-etching and rotary-shadow replication (QF-FF-DE-RR) revealed special details in structures, such as subpellicular microtubules, the kinetoplast and the flagellum, a structure that, in trypanosomatids, is formed by the axoneme and the paraflagellar rod (PFR) [2].

A typical flagellum is formed by an axoneme composed of a 9+2 system of microtubule doublets enclosed by the flagellar membrane. A complex system of short projections made of protein complexes form the outer and inner arms of the peripheral doublets and the radial spokes. In addition, protein complexes forming the intraflagellar particles can also be seen between the peripheral microtubule doublets and the flagellar membrane [3]. The flagella of trypanosomatids present another structure known as the paraflagellar rod (PFR), which is a filamentous structure that runs over the length of the flagellum along the axoneme of most of the kinetoplastid flagellates [4]–[7]. Unlike the axoneme, which is conserved among eukaryotes, the PFR is restricted to kinetoplastids, euglenoids and dinoflagellates and is important for cell motility [8]–[9] and for the attachment of the parasite to specific tissues in the insect vector [10]–[12]. The analysis of thin sections of detergent-extracted and glutaraldehyde-tannic acid-fixed cells as well as replicas made using the QF-FF-DE-RR technique showed that the PFR is organized by discrete filaments structured in lattice-like arrays with three distinct domains: proximal, intermediate and distal [13]. Each domain is formed by a complex array of 25-nm thick filaments intercrossed by 7-nm filaments at an angle of 100° [7], [13].

Biochemical analysis of the PFR allowed the description of two major proteins (PFR1 and PFR2) and several minor components [14]–[16]. A novel myosin isoform, myosin XXI, was also localized in an area related with the PFR, especially at the basal portion of the flagellum [17]. However, we still do not have a clear understanding of the relationship between the nature of the protein and the filaments that compose the PFR.

Recent studies using approaches such as the silencing of genes involved in the assembly of the PFR in *Trypanosoma brucei* [5] or gene knockout of PFR proteins in *Leishmania mexicana* [18], which led to partial or total impairment of motility, have indicated that the PFR plays some role in flagellar movement [19]–[20].

Atomic force microscopy (AFM) is a powerful tool to provide high-resolution images and has been used for several years in biological sciences. A few attempts to analyze the structural organization of pathogenic Protozoa have been performed using AFM [21]–[22]. Our group has recently used AFM to re-visit the ultrastructure of the flagellum of *T. cruzi* [23], and novel structural features, such as the presence of a periodic array of particles, were observed along the flagellum of this protozoan, although their functional role is still unknown due to the lack of a detailed molecular and biochemical characterization.

The dynamics of flagellar beating in trypanosomatids involve intense conformational changes of the axonemal microtubules and associated structures and, presumably, the PFR filaments. Previous studies using deep-etching replicas have provided high-resolution images of the flagellar region, including the PFR [7], [13]. The main goal of these studies was to reveal the structural organization of the *T. cruzi* flagellum, and they did not take into account if the analyzed flagella were in straight or bent states. This was mainly due to the fact that random fractures were obtained from ice-embedded samples, making the precise determination of the three-dimensional (3D) conformation of the flagella a challenging task. With AFM, it is relatively easy to determine the flagellar bending state and image different portions of its structure.

In the present study, we analyzed the sub-structure of the PFR and flagella in straight or bent states at high resolution by AFM and TEM of replicas of QF-FF-DE-RR epimastigote forms of *T. cruzi*. Images obtained using both techniques revealed the spatial distribution of PFR filaments at high resolution (those filaments located on the top and bottom of each array that composes the whole structure) and their association with the filaments of the axoneme. Measurements of the distances between PFR filaments, axoneme components and the filaments that connect them suggested that changes in the angles between the intercrossed filaments of the intermediate domain of the PFR during flagellar beating might induce a remodeling of the whole structure. Overall, the images obtained suggest that a sliding of the intermediate PFR filaments takes place during flagellar beating. Using graphic computation based on those measurements, we propose a hypothetical model that suggests that the three portions of the PFR are rearranged, undergoing a dynamic remodeling during flagellar beating.

## Results

### SEM and TEM of thin Sections

**Figure 1:**
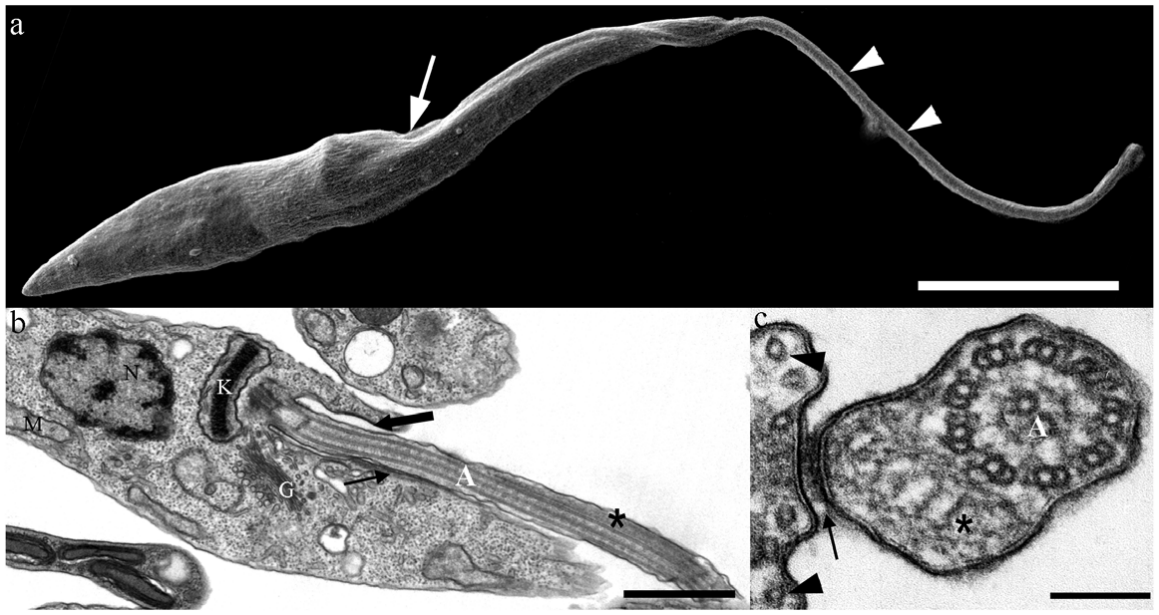
Electron micrographs of epimastigote form of *T. cruzi*. (a) Scanning electron micrograph of *T. cruzi*. The flagellum (arrowheads), which emerges from the flagellar pocket (arrow), is attached to the cell body along the flagellar attachment zone (FAZ) region. (b–c) Transmission electron micrographs of ultrathin sections of epimastigote forms. The axoneme (A) and paraflagellar rod (*) are seen in both longitudinal (b) and transversal (c) sections. In longitudinal sections, it is clear that the flagellum emerges from the flagellar pocket (thick arrow). FAZ region (arrows) and subpellicular microtubules (arrowheads in c) can also be observed. (N) Nucleus, (K) kinetoplast, (G) Golgi complex, (M) mitochondria. Bars: a – 5 µm; b – 1 µm; c – 100 nm.

Figure 1a shows a general view of the epimastigote form of *T. cruzi* as seen using SEM. The area of emergence of the flagellum, its attachment to a portion of the protozoan body and the free flagellum are illustrated. TEM of thin sections at the areas indicated in Fig. 1a shows the emergence of the flagellum from the flagellar pocket and its initial association with the cell body. In this view (Fig. 1b); it is possible to identify the flagellar axoneme and the presence of the PFR. In a cross section of the flagellum (Fig. 1c), it is possible to clearly visualize the presence of the PFR.

### AFM images

**Figure 2:**
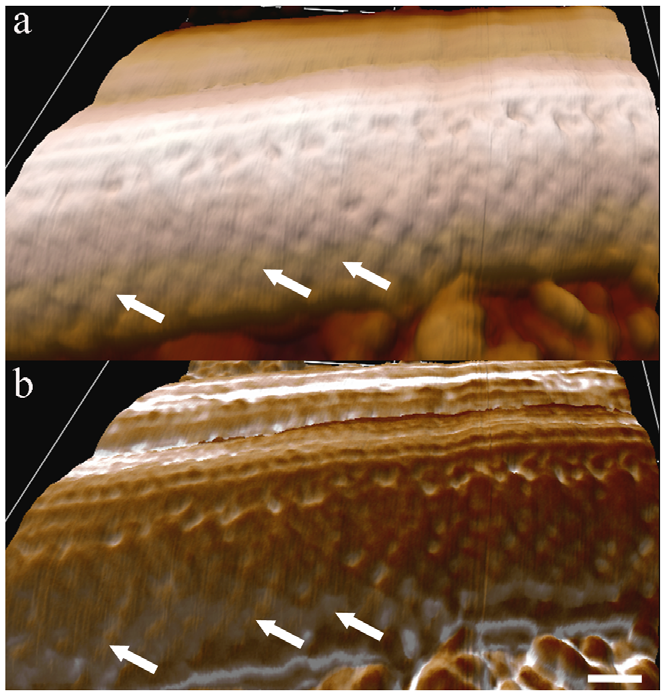
AFM intermittent contact mode image of the flagellum of *T. cruzi* epimastigote form. (a) Topographic 3D view of the flagellum with smooth information of PFR filaments (arrows) detected. (b) Combined topographic and phase signals of the same region shown in (a). Bar – 100 nm.

Topographical AFM intermittent contact mode images of the flagellar region (Fig. 2a) of whole cells revealed details, such as the flagellar surface on which periodic strands that compose the PFR were identified, especially when phase and height signals were combined (Fig. 2b).

**Figure 3:**
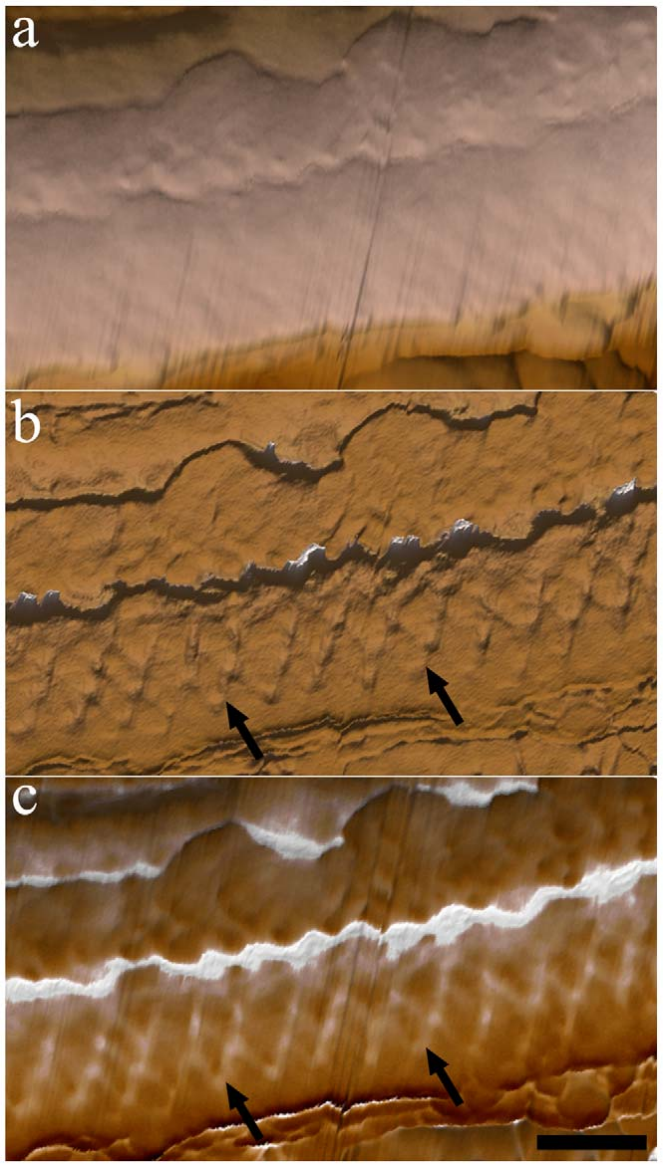
AFM intermittent contact mode image of straight state flagellum of detergent-extracted epimastigotes. (a) Topographic 3D view of part of the flagellum. Note that information on the PFR filaments is almost undetectable. (b) Phase image of the flagellum showing the lattice organization of the filaments of the PFR (arrows). (c) Combined topographic (a) and phase (b) signals. Bar – 200 nm.

**Figure 4:**
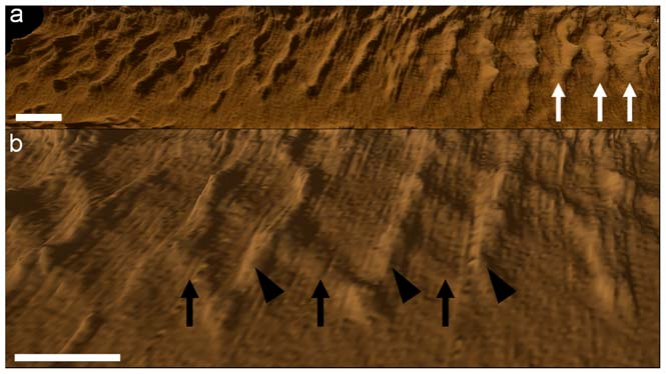
AFM phase signal images of the flagellar region of detergent-extracted epimastigotes. (a) In the right region of the image, in which the flagellum starts to bend, the PFR signal is more pronounced (white arrows). (b) Top (arrowheads) and bottom (black arrows) positions of PFR intercrossed filaments (those that lie over each other) are observed. Bars – 100 nm.

AFM images of detergent-extracted cells revealed structures that were not easily visualized by TEM of thin sections. Figure 3 shows (a) height and (b) phase signals of a detailed portion of a flagellum in a straight state in which the network of intercrossed filaments that forms the PFR can be observed, especially when phase signals are displayed (Fig. 3 b–c, arrows). Morphometric analysis showed that these filaments are approximately 12 nm thick and are spaced by a distance of 60 nm in straight flagellar state. Combined height and phase signals are shown in Fig. 3c. In some instances, the relative position of the top (arrowheads) and bottom (white arrows) rows of PFR filaments of the intermediate domain could be observed (Fig. 4b). Changes in the properties of the sample surface alter the pattern of phase signals. Figure 4a shows a single flagellum but allows for the observation of one segment in a straight state (left side of the image) and another segment in a bent state (right side). Phase signals are more intense in the region in which the flagellum is in a bent state (Fig. 4a, black arrows) when compared to a straight state, probably due to differences in the tensions in the PFR filaments between these two regions.

**Figure 5:**
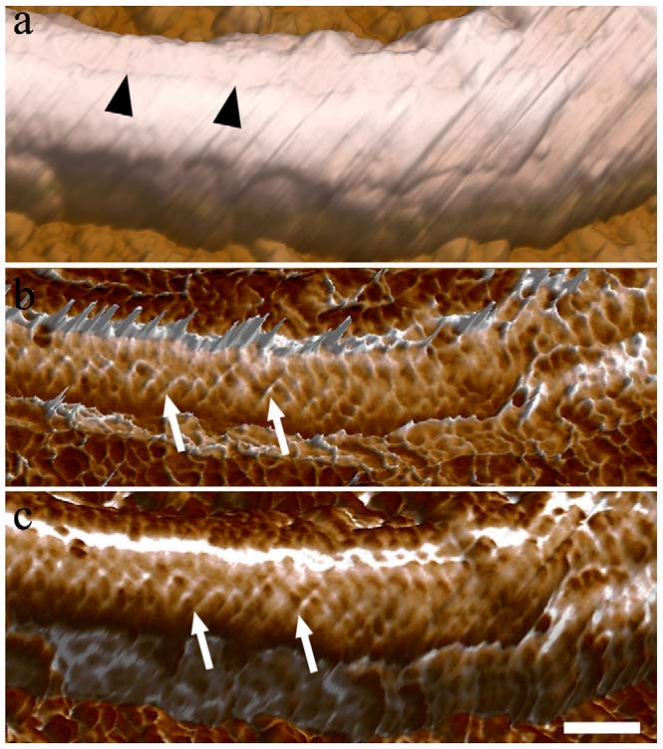
AFM intermittent contact mode image of bending state flagellum of detergent-extracted epimastigote forms. (a) Topographic 3D view of the PFR structure. The furrow can be observed (arrowheads). (b) Phase image of the flagellum in a bending state shows the distribution of the filaments of the PFR (arrows) along the flagellum. (c) 3D view of combined height and phase images. Bar – 200 nm.

Figure 5 shows AFM images of portions of the flagellum in a bending state. A topographic 3D view (height signals) shows a furrow that is present in the region between the PFR and the axoneme (Fig. 5a). As in Fig. 3, phase images (Fig. 5b) better revealed the network of intercrossed filaments that forms the PFR. Morphometric analysis showed that the thickness of these filaments in bending states did not alter in relation to the filaments in straight states, which are approximately 12 nm thick. Moreover the filaments are closer to each other by a distance of 45.23 nm in bending state flagellum. Measurement analysis of the filaments of the intermediate domain of the PFR showed that the angles of intercrossed filaments are higher when compared to flagella in a straight state. Quantitative data on the morphometric parameters of the flagellum in different bending states are summarized in Table 1.

**Table 1:** Morphometric analysis of intercrossed filaments of the intermediate domain of flagella in different bending states.

Bending states	Angles (°)AFM	Angles (°)Deep-etching
Low curvature	74±0.7	85±0.5
Medium curvature	81±0.7	100±0.8
High curvature	98±0.8	112±1.3

Comparison between deep-etching replicas and AFM data. Results are expressed as mean ± standard deviation.

### TEM imaging of deep-etching replicas

**Figure 6:**
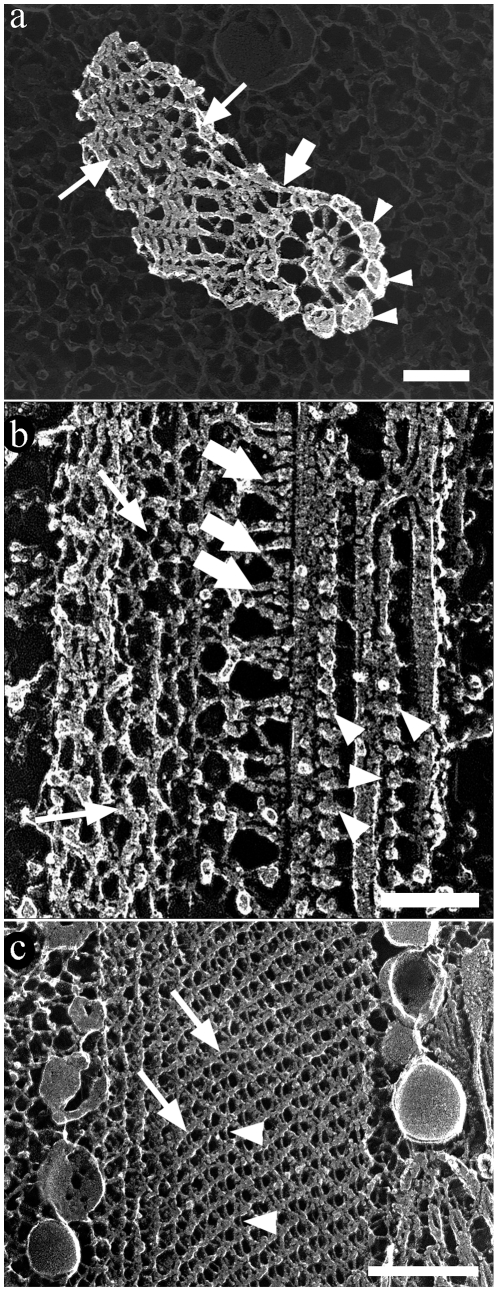
Deep-etching replica images of the flagellum of *T. cruzi* epimastigote form. The main components of the flagellum are clearly shown in transversal (a) and longitudinal (b–c) fractures. (a) The peripheral (arrowheads) as well as the central pair of microtubules of the axoneme are seen. The PFR is seen in the left side of the figure (arrows). (b) Transversal fracture of the flagellum shows microtubules of the axoneme, dynein units (arrowheads) and the PFR (arrows). The flagellum is in a straight state with the PFR in a relaxed state. (a–b) The axoneme and the PFR are linked by fibers indicated by thick arrows. (c) Deep-etching replica image of the PFR with intercrossed thicker (arrows) and thinner filaments (arrowheads). These images were digitally processed in order to emphasize only the components of the flagellum against a shallow background. Bar – 100 nm.

TEM of replicas of QF-FF-DE-RR provided high-resolution images of the main components of the *T. cruzi* flagellum. Using this technique, in several images recorded (approx. 16) it was possible to clearly distinguish (a) the axoneme, seen both in transversal (Fig. 6a) and longitudinal (Fig. 6b) fractures, and associated dynein units (Fig. 6b, arrowheads) that appear, as previously well characterized in the flagellum of *Chlamydomonas reinhardtii* [24], as a structure made by globular components measuring 23.3×15.9 nm supported by a 5.0-nm thick arm with a length of 18.7 nm. It was also possible to clearly distinguish (b) the 7.1-nm thick and 57.1-nm long single filaments connecting the axoneme to the proximal portion of the PFR that periodically alternate with 4.7-nm thick filaments, forming a “Y”-like structure (Fig. 6b, thick arrows) that form a 53.2° angle in a straight state and a 41.3° angle in a bent state. Short (58.2 nm) and long (63.0 nm) “Y” filaments were found: (c) the filamentous array of the proximal and distal portions of the PFR and (d) the filamentous array of the intermediate portion, formed by 9.9±0.94-nm and 4.2±0.53-nm thick filaments (Fig. 6c). These filaments formed a lattice-like structure (Fig. 6b–c, thin arrows), as the filaments that formed the proximal domain (Fig. 6 a–b, thick arrows). Measurements of these filaments are summarized in Table 2.

**Table 2:** Morphometric analysis of the filaments that compose the flagellar structure.

	Short diameterof globular domain	Large diameterof globular domain	Arm thickness	Arm length
Dynein	15.9±0.70	23.3±0.98	5.0±0.30	18.7±0.86	

	Single filamentsthickness	Single filamentslength	Double “Y”filaments thickness	Large “Y”filament length	Short “Y”filament length
Filament connectionsBetween the PFRand the axoneme	7.1±0.38	57.1±2.37	4.7±0.59	63.0±2.93	58.2±2.93

Data was obtained from deep-etching replica images. Results are expressed as mean ± standard deviation (nm).

**Figure 7:**
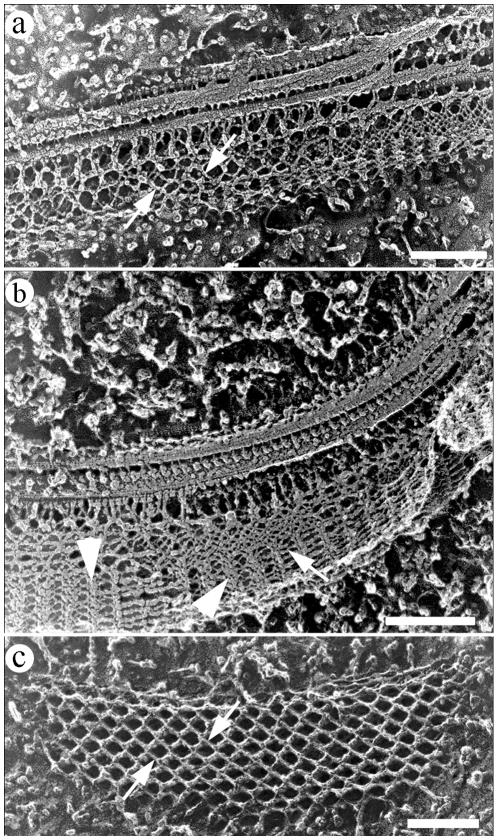
Deep-etching replica of flagellar regions in different states: straight (a) and bent (b). Angles between PFR filaments (arrows) vary according to the bending state of the flagella. (c) Deep-etching image showing a longitudinal fracture of the intermediate domain of the PFR in a flagellum presumably in a straight state. Band-like structures on (b) are indicated by arrowheads. Bars – 250 nm.

Deep-etching replicas of flagella in straight (Fig. 7a) and bent (Fig. 7b) states showed the PFR at different degrees of structural adaptation to the curvature of the axoneme. As in AFM preparations, changes in the angles between the filaments of the intermediate domain of the PFR (Fig. 7c) as a function of the curvature of the flagella were also seen in deep-etching images. In a straight or low curvature state, the angles were limited to approximately 85°. At high curvatures, when the whole structure seemed to be more compact (Fig. 7b), these filaments tended to be closer to each other and achieved a maximum angle of 112°, even revealing some structures that appeared as bands formed by PFR filaments.

Quantitative data on the morphometric parameters collected from deep-etching images of the flagellum in different bending states are summarized in Table 1. The distances between parallel filaments and areas contained inside the perimeter formed by two pairs of intercrossed filaments were measured as a parameter for packed or loose states of the filaments of the intermediate domain. Considering that the loosest conformation of the filaments defined a geometric form close to a square (Fig. 7c), a square shape factor was also defined (see materials and methods) as a parameter for packed or loose states in which 1.0 defines a perfect square (loose). Results revealed that distances between the parallel filaments and the shape factor decreased with increasing curvature of the flagellum (Table 3), indicating a packed configuration of the PFR in bent states.

**Table 3:** Comparison of intercrossed filaments of intermediate domain in different flagella bending states in deep-etching replicas.

Bending states	PFR filamentsdistance (nm)	PFR filamentsdistance (nm)	Shape Factor
Low curvature	46.3±4.36	45.3±5.86	1
Medium curvature	50.4±4.31	44.7±2.17	0.91
High curvature	54.5±4.55	12.9±1.54	0.60

Results are expressed as mean ± standard deviation.

## Discussion

The PFR found in some protists is an excellent example of assembly of proteins into filamentous structures that then interact with other filaments to form more complex assemblies. A tremendous amount of information is available on the structure, composition and functional role played by the flagellar axoneme [9]. However, there is still little information on the structure, composition and function of the PFR, which is an intrinsic component of the flagellum of most trypanosomatids. Previous structural analysis has contributed to the current knowledge on the structural organization of the PFR. Most of the information comes from TEM studies of thin sections of glutaraldehyde-tannic acid-fixed cells or of replicas obtained using the QF-FF-DE-RR technique [7], [13]. The present study combines the use of AFM and TEM, revealing some new information on the organization of the PFR, especially of its intermediate region. In contrast, the proximal and distal domains are not so easily visualized using AFM, probably due to the orientation of the scanning line and the nature of the cantilever, which make the analysis of these domains almost impossible.

The QF-FF-DE-RR technique, established by Heuser and co-workers [25], has been used to analyze the flagellum of trypanosomatids. This technique allows the visualization of the whole structure, including the connection of the PFR to the axoneme.

In a previous study, we used AFM to analyze basic aspects of the fine structure of *T. cruzi* [23]. Our present observations show that AFM may also be used to further analyze the sub-structure of the PFR. There are many AFM scan modes. Among them, the phase imaging mode provides both topographical information and the detection of adhesive and elasticity properties. This mode of imaging allows for a better visualization of structures, such as the PFR, and a more precise identification of which type of filaments lie over each other.

The association of images obtained using freeze-fracture and deep-etching with AFM height and phase images allowed for a good comparison between the two techniques. From phase AFM images, a kind of 3D view allowed the visualization of the intercrossing of the PFR filaments, while a 2D view was obtained in freeze-fractured and deep-etching replicas.

### Flagellar beating and PFR conformational change

There is no previous comparative analysis of the structural organization of the PFR of trypanosomatids in flagella in straight versus bent states. A comparative analysis using deep-etching and AFM images of the PFR filaments provided information on how the PFR changes its conformation in different flagellar bending states.

In 1972, Bovee and Jahn [26] proposed the theory that the axoneme and PFR would be composed of asymmetrically crystalline proteinaceous fibrils that have piezoelectric activity, triggering sequential bending and straightening of the segments of the flagellum from base to tip. In the present report, we speculated that the PFR has a dynamics, according to the movement of the axoneme. We have seen not only a change of direction of the PFR filaments but also that these filaments have higher tensile strength during flagellar bending as measured using the phase imaging mode, therefore agreeing with the theory proposed by [26].

We observed that when the flagellum is in a straight state, PFR intermediate filaments are further apart from each other. These filaments begin to get closer at the beginning of flagellar bending, and this phenomenon is more progressive when bending increases. In a higher bending state, as shown in Fig. 7c, structures displaying a “band”-like appearance were seen, probably due to the fact that the filaments of the PFR remained closer to each other, giving the impression that this aggregate of filaments forms a “band” structure.

**Figure 8:**
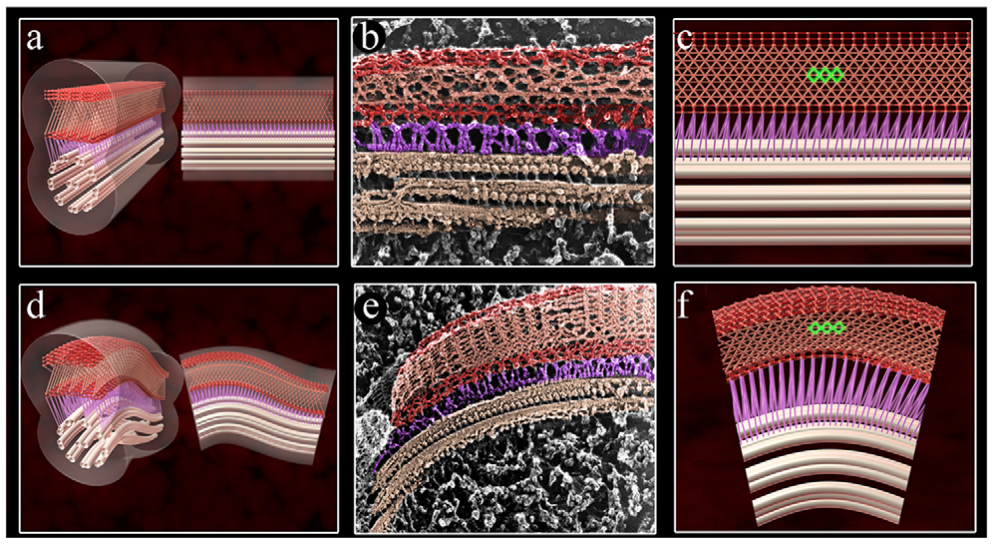
Frame view of PFR animation during flagellar beating in comparison with deep-etching replicas. In a straight state (a–c), the intercrossed filaments reveal a regular diamond structure (highlighted in green in c). Panels d-f show the flagellum in a bent state and the intercrossed filaments in a different transition relative to the flagellum in a straight state. (f) An irregular diamond structure can be observed (highlighted in green). Deep-etching replicas images of the flagellum in straight (b) and bent (e) state. Note that the flagellar structures in replicas were colored in the same pattern of the animation. Axoneme – light pink, filaments that link the PFR to the axoneme – purple, proximal and distal domains of the PFR – red and the intermediate domain – salmon.

To obtain more details of the PFR structure, we pooled all obtained information into an animation. Supplemental videos S1 and S2 simulate how the PFR changes its filaments during the beating process. A stationary frame of the flagellum in straight and bent states is observed in figure 8. It is possible that the intercrossed filaments of the intermediate domain move in opposite directions to each other. At a higher curvature of the flagellum, the thickness of the PFR would decrease because the filaments change direction during flagellar beating. In addition, based on the possible movement of the intermediate domain, our model suggests that the proximal and distal domains probably have the same particularity of movement as the intermediate domain, even considering that we do not have as much information on these domains. Our hypothesis is that these three domains work together, and in a moment when one domain, e.g., the proximal domain, is compressed, the distal and opposite domain will be stretched. This movement would alternate during flagellar beating. The intermediate domain would follow this dynamic of movement, and in a bent state, their filaments would get closer. At present we have no information about the molecular basis of the PFR movement. One possibility, which deserves further analysis, is the participation of the recently described myosin XXI isoform in this process [16]–[17].

Moreira-Leite and co-workers [15] showed many minor components of the PFR and suggested that one of these could be a motor protein that would open the possibility for the intermediate domain to change its position between proximal and distal filaments. At present, the nature and function of these proteins are unknown. It is probable that each contact point of the filaments of the intermediate domain possesses a domain that allows movement and provides a point of anchoring to the filaments that may have a degree of distention. Further biochemical studies could determine the type of protein connection between the filaments.

Altogether, our present observations show AFM as a powerful technique that can provide excellent information, especially when combined with complementary EM methods, such as deep-etching images, and help to understand the dynamic remodeling of the PFR during flagellar bending of trypanosomatids.

## Materials and Methods

### Parasites

Epimastigote forms of *T. cruzi* (Y strain) were cultivated in liver infusion tryptose (LIT) medium supplemented with 10% fetal bovine serum and 1% hemin for 3–5 days at 28°C.

### Scanning electron microscopy

To prepare cells for scanning electron microscopy (SEM), cells were fixed in 2.5% glutaraldehyde in 0.1 M phosphate buffer, pH 7.2, for 60 minutes, washed in the same buffer and deposited onto poly-L-lysine-coated coverslips. Cells were then post-fixed in 1% OsO_4_ and 0.8% potassium ferrocyanide in 0.1 M sodium cacodylate buffer, pH 7.2, at room temperature for 30 minutes, washed in 0.1 M phosphate buffer, dehydrated in an ethanol series, and critical point dried with CO_2_. Samples were ion-sputtered with a 15 nm chrome layer using a Leica model EM SCD 500 to avoid charge effects. Secondary electron images were obtained using a Nova 600 Nanolab Dual Beam at a 2.0 kV accelerating voltage.

### Transmission electron microscopy

Cells were fixed in 2.5% glutaraldehyde in 0.1 M phosphate buffer, pH 7.2, for 60 minutes, washed in the same buffer, post-fixed in 1% OsO_4_ and 0.8% potassium ferrocyanide in 0.1 M sodium cacodylate buffer at room temperature for 40 minutes, washed in 0.1 M phosphate buffer, dehydrated in acetone, and embedded in Polybed resin. Ultrathin sections were stained with uranyl acetate and lead citrate and observed using a Zeiss EM 900 transmission electron microscope.

### Atomic force microscopy

Epimastigote forms were collected by centrifugation (1100 g for 15 minutes at room temperature), washed in 0.1 M phosphate buffer, fixed in 2.5% glutaraldehyde in 0.1 M phosphate buffer, pH 7.2, for 60 minutes, washed in the same buffer, deposited onto poly-L-lysine-coated glass coverslips, dehydrated in an ethanol series and critical point dried with CO_2_. For the extraction of the plasma membrane, the parasites were washed for 10 minutes with 0.1 M PHEM buffer [60 mM 1,4 piperazine diethylsulfonic acid (PIPES), 25 mM N-2-hydroxyethylpiperazine N1-2-ethanesulfonic acid (HEPES), 10 mM EGTA, and 2 mM MgCl_2_], [27], pH 7.2, and then deposited onto poly-L-lysine-coated glass coverslips. The plasma membrane was extracted with 1% NP-40 in 0.1 M PHEM buffer for 5–7 minutes and immediately fixed with 2.5% glutaraldehyde in the same buffer for 2 minutes. Samples were then washed twice in distilled water and dried using a low jet of argon gas. Cells were examined in an MFP-3D atomic force microscope (Asylum Research, Santa Barbara – CA). The glass slide containing the sample was mounted onto the XY scanner of the AFM, and a CCD camera was used to localize the parasites.

Images were acquired in air using contact and intermittent contact modes. Cantilever elastic constants were obtained using the thermal noise method. For contact mode imaging, V-shaped standard narrow cantilevers, model NP-S (Veeco Probes, Camarillo, CA), were used. The spring constant was typically 0.08 N/m, and samples were scanned at constant force with a low scan rate (0.6 Hz) in order to reduce noise and minimize sample damage. For intermittent contact mode, tetrahedral-shaped cantilevers (AC240TS, Olympus, Tokyo, Japan – nominal spring constant 2 N/m) were used at a low scan rate (0.5 Hz). Images were acquired with 512×512 pixels of resolution, and image processing (line-wise flatten only) was performed in IGOR-PRO (Wavemetrics, Portland, OR) using an MFP-3D template developed by Asylum Research. For better topographic visualization, height images were displayed as 3D views using the ARgyle Light program developed by Asylum Research to obtain an overlay of the topography with the phase signal obtained.

### Quick-freezing, freeze-fracture, and deep-etching

Epimastigote forms were briefly fixed in 2.5% glutaraldehyde in 0.1 M phosphate buffer, pH 7.2, for 60 minutes, washed with 0.1 M phosphate buffer, pH 7.2, and rinsed in Milli-Q water. Cells were then mounted on aluminum support disks and slammed onto a liquid helium-cooled copper block of a quick freezing device (Cryopress Med-Vac). Fracture was carried out at −115°C in a Balzers apparatus. After fracturing, the temperature was raised to −105°C for 10–20 minutes for etching. Platinum was evaporated onto the specimen at an angle of 15° as the specimen was rotated, and carbon was then evaporated at an angle of 90°. Replicas were cleaned to remove the remaining organic material by digestion with sodium hypochlorite, rinsed with distilled water, mounted on 300-mesh nickel grids and observed in a Zeiss EM900 transmission electron microscope. Micrographs were examined in negative contrast by photographically reversing them before printing to make the platinum deposits appear white and the background appear dark. As previously shown [25], this contrast reversal enhances the 3D appearance of the images. In some images, specific structures were digitally highlighted for better visualization of the flagellar components.

### Morphometric analysis

Freeze-fractured, deep-etching and AFM images of the flagellar region of epimastigote forms of *T. cruzi* were selected, and the distance between filaments of the PFR oriented relative to the major axis of the axoneme was measured. Measurements were taken from the center of one filament to the center of its neighbor. The angles between intercrossed filaments were also measured. A square shape factor (S) was defined by dividing values of the area and perimeter formed by two pairs of intercrossed filaments according to the formula: S = 16×A/P^2^, where 1.0 defines a perfect square. ImageJ was used to measure these angles. To get data to the morphometric analysis, sixteen (16) flagellar sections from freeze-fractured and deep-etching replicas and twelve (12) profiles of the flagellar region from AFM images were used.

### Generation of 3D models and animation movies

The 3D models and animations based on the images obtained using AFM and TEM of replicas of quick-frozen, freeze-fractured and deep-etched cells were obtained using Max, Maya, and Flash programs. Final movies from the individual sequences of images were rendered in 3Ds Max and Maya programs and encoded in FLV (Flash Video) format (Flash Player; http://get.adobe.com/flashplayer/).

## Supporting Information

Video S1 Animation of flagellar beating of the PFR in a longitudinal view. This animation is simulating how the PFR filaments change during the beating process. This video allows a better visualization of the intercrossed filaments of the intermediate domain moving in opposite directions to each other.
(2.68 MB MP4)

Video S2 Transversal view of PFR animation during flagellar beating. The proximal and distal domains have a same peculiarity of intermediate filaments movement.
(2.67 MB MP4)
